# Human pathological findings in Kawasaki disease: a narrative review of autopsy and biopsy evidence

**DOI:** 10.3389/fimmu.2026.1798378

**Published:** 2026-05-07

**Authors:** Jiaying Zhang, Junru Chen, Yinghao Wang, Ying Liu, Jing Li, Difan Wang, Panpan Liu, Zhiyuan Liu, Mingyang Zhang, Tingjiao You, Qiuyu Tang, Chengyi Wang, Haitao Lv, Hongbiao Huang

**Affiliations:** 1 Department of Cardiology, Children’s Hospital of Soochow University, Suzhou, Jiangsu, China; 2 Institute of Pediatric Research, Children’s Hospital of Soochow University, Suzhou, Jiangsu, China; 3 Pediatric Intensive Care Unit, Fujian Children’s Hospital, Fujian Branch of Shanghai Children’s Medical Center, College of Clinical Medicine for Obstetrics & Gynecology and Pediatrics, Fujian Medical University, Fuzhou, Fujian, China; 4 Pediatric Infectious Diseases Department, Fujian Children’s Hospital, Fujian Branch of Shanghai Children’s Medical Center, College of Clinical Medicine for Obstetrics & Gynecology and Pediatrics, Fujian Medical University, Fuzhou, Fujian, China; 5 Department of Pediatrics, Fujian Provincial Hospital, Fuzhou University Affiliated Provincial Hospital, Fuzhou, Fujian, China

**Keywords:** autopsy, biopsy, coronary artery lesions, Kawasaki disease, molecular mechanisms, pathology, vasculitis

## Abstract

**Background:**

Kawasaki disease (KD) is an acute systemic vasculitis affecting children under five years of age and a leading cause of acquired heart disease in developed countries. Although autopsy and biopsy studies provide important insights into disease progression, integrated summaries combining classical histopathology with modern molecular findings remain limited.

**Methods:**

This narrative review searched PubMed and Web of Science from January 1, 1974 to December 31, 2025 for human autopsy and biopsy studies on KD. Findings were synthesized to characterize pathological features across cardiovascular and extracardiac systems, focusing on vascular progression based on the three-process model, multisystem involvement, and underlying molecular and genetic mechanisms.

**Results:**

Coronary artery involvement follows a three-process model, including necrotizing arteritis, subacute/chronic vasculitis, and luminal myofibroblastic proliferation, which drives progressive luminal stenosis. The myocardium, pericardium, and cardiac valves are also frequently affected. Extracardiac tissues show intracytoplasmic inclusion bodies and IgA plasma cell infiltration, suggesting a potential infection-triggered mechanism. Key signaling pathways, including TLRs/NF-κB, NLRP3/IL-1β, Ca²^+^/NFAT, and TGF-β, along with genetic polymorphisms, contribute to immune dysregulation and vascular injury and may provide potential therapeutic targets.

**Conclusion:**

KD is a systemic vasculitis primarily targeting the coronary arteries, with a dynamic pathological progression from acute inflammation to chronic vascular remodeling. Future research should focus on prospective pathology studies, long-term vascular remodeling, and underlying molecular mechanisms.

## 1 Introduction

Kawasaki disease (KD), originally termed “acute febrile mucocutaneous lymph node syndrome”, was first described by Tomisaku Kawasaki in 1967, with the first English-language report published in Pediatrics in 1974 (1, 2). KD is an acute systemic vasculitis of unknown etiology that predominantly affects children under 5 years of age, with the highest incidence observed in East Asian populations (3). The diagnosis of KD is mainly based on a set of characteristic clinical criteria, including persistent fever (≥5 days) accompanied by at least four of the following principal features: bilateral bulbar conjunctival injection without exudate; changes in the lips and oral cavity (such as strawberry tongue, erythema and cracking of the lips, and diffuse erythema of the oropharyngeal mucosa); polymorphous rash; changes in the peripheral extremities (including indurative edema of the hands and feet in the acute phase and membranous desquamation of the fingertips or toes during the convalescent phase); and cervical lymphadenopathy (typically unilateral, ≥1.5 cm in diameter) (4, 5). These clinical manifestations reflect the systemic inflammatory nature of the disease. Although the acute inflammatory phase of KD is generally self-limited, involvement of medium-sized muscular arteries, particularly the coronary arteries, can result in vasculitis with serious clinical consequences (6). Coronary artery aneurysm (CAA) formation is the most significant complication and may lead to thrombosis, acute myocardial infarction (AMI), and sudden cardiac death (7). Timely administration of high-dose intravenous immunoglobulin (IVIG) can markedly reduce the incidence of CAA; however, approximately 10–20% of patients remain resistant to treatment and are consequently at increased risk of adverse outcomes (4).

Pathological studies are essential for understanding the severe complications of KD and for providing insights into its etiology, thereby informing clinical management. Early autopsy studies established KD as a systemic vasculitis, while more recent investigations, using advanced techniques such as immunohistochemistry, electron microscopy, and molecular analyses, have further elucidated the complex cellular mechanisms underlying vascular wall remodeling and provided evidence supporting a potential infectious contribution (8, 9). Previous reviews on KD have primarily focused on etiology, immunopathogenesis, epidemiology, diagnostic and therapeutic strategies, as well as genetic susceptibility (10–13). In addition, some studies have incorporated pathological findings based on autopsy data, mainly describing histopathological features associated with cardiovascular complications and sudden death (14). Autopsy-based studies have identified coronary artery damage, vascular remodeling, and myocardial fibrosis as key long-term pathological changes in KD (7). However, a comprehensive synthesis integrating human autopsy and biopsy evidence across multiple organ systems is still lacking.

To address this gap, the present narrative review aims to provide a synthesis of histopathological evidence in KD. A literature search was conducted using databases including PubMed and Web of Science, covering studies published from January 1, 1974 to December 31, 2025. The search was performed using combinations of keywords, including “Kawasaki disease”, “mucocutaneous lymph node syndrome”, “pathology”, “autopsy”, “biopsy”, and “vasculitis”. Eligible studies focused on human histopathological findings derived from autopsy or biopsy specimens, encompassing both classical morphological observations and more recent advances in molecular pathology. The retrieved evidence was qualitatively synthesized to characterize the progression of vascular injury based on the three-process model, to describe cardiovascular and extracardiac involvement, and to integrate molecular mechanisms of vascular remodeling within the immune-inflammatory microenvironment.

This multidimensional integrative perspective aims to provide an updated pathological framework that may improve understanding of the systemic nature of KD and help identify potential pathogenic targets and inform long-term prognosis.

## 2 Main pathological features of Kawasaki disease

KD is characterized by systemic vasculitis involving small- and medium-sized arteries. The severity and progression vary across individuals and vascular beds, and it primarily affects the coronary arteries. Although acute inflammation is typically self-limited, some patients develop serious cardiovascular complications, including CAA, AMI, heart failure, arrhythmia, and acute coronary syndrome, which are major causes of mortality (14). Autopsy and biopsy studies further indicate that small- to medium-sized arteries in other organ systems, such as the respiratory, digestive, and urinary systems, may also exhibit inflammatory infiltration.

Although significant progress has been made in the clinical diagnosis and treatment of KD, pathological evidence obtained through autopsy remains fundamental for understanding its pathogenesis and disease evolution. The currently available histopathological evidence can be broadly categorized into three main components: classic autopsy studies from the pre-IVIG era, reassessment of historical samples using modern techniques, and rare autopsy cases of long-term cardiovascular sequelae in KD patients. **Supplementary Table 1** summarizes human pathological studies on KD from early to contemporary research.

### 2.1 Coronary artery and systemic vasculitis (the three-process model)

Based on light microscopy and transmission electron microscopy (TEM) studies, contemporary pathology has proposed the “three linked vasculopathic processes” model of KD vasculopathy. This model suggests that KD vasculitis involves three distinct yet interrelated pathological processes (**Supplementary Table 2**) (8).

#### 2.1.1 Necrotizing arteritis

NA is characterized by a highly synchronized infiltration of neutrophils occurring within the first two weeks after fever onset. The process begins in the vascular endothelium and progresses outward layer by layer, sequentially destroying the intima, internal elastic lamina (IEL), media, and external elastic lamina (EEL), and may extend to the adventitia (8). This process results in severe disruption of the vessel wall architecture and represents the primary mechanism underlying saccular aneurysm formation (8). Histological examination shows that the necrotic vessel wall consists of a mixture of neutrophils, cellular debris, and fibrin. Although NA is the only self-limited process in the course of KD, it may, in some cases, serve as a key pathological basis for early aneurysm rupture or acute thrombosis, ultimately leading to sudden death (8, 14). This vulnerability is attributable to the extremely thin residual aneurysmal wall, in which the adventitia, pericardium, and perivascular tissue are typically less than 1 mm in thickness; in some cases, the aneurysmal wall consists only of a small amount of pericardial or perivascular tissue (8). An autopsy report of an infant who died approximately 15 days after disease onset revealed transmural inflammatory cell infiltration of the coronary artery wall, resulting in structural destruction and aneurysm formation, with fresh thrombus observed within the lumen (15).

#### 2.1.2 Subacute/chronic vasculitis

SA/C vasculitis is characterized by an asynchronous process of inflammatory infiltration (8). This process typically begins approximately two weeks after fever onset but may persist for months or even years in a subset of patients. The inflammatory infiltrate is predominantly composed of lymphocytes, plasma cells, and eosinophils, with a smaller proportion of macrophages (8). The process generally originates in the adventitia or perivascular tissues and progresses inward toward the lumen, leading to varying degrees of vascular inflammation and structural damage (8). In addition to contributing to aneurysm formation and persistence, SA/C vasculitis promotes the transformation of smooth muscle cells (SMCs) into myofibroblasts, leading to progressive luminal stenosis (8).

#### 2.1.3 Luminal myofibroblastic proliferation

LMP is distinct from simple scar repair or thrombus organization and represents a unique process driven by myofibroblasts derived from medial SMCs (8, 16). It typically begins around two weeks after fever onset and may persist for months to years. LMP drives progressive intimal hyperplasia and is a key pathological mechanism underlying coronary artery stenosis and occlusion in the late stage of KD (8). Under the influence of SA/C inflammation, these myofibroblasts migrate and proliferate, leading to asynchronous and progressive luminal narrowing (8). Autopsy studies of patients who died from late KD sequelae frequently show thrombus organization and recanalization, extensive calcification, and marked fibrotic intimal thickening associated with LMP (**Table 1**) (7, 17). Notably, vasculitis of the coronary arteries has been observed even in patients without CAA at autopsy, including those without coronary artery dilation during the acute phase. Moreover, studies indicate that even when coronary dilation appears to regress on imaging, the underlying pathology often reflects persistent fibrotic intimal thickening rather than true normalization of the vessel wall (18).

**Table 1:** Autopsy findings of long-term cardiovascular sequelae in KD.

Reference	Sample size (N)	Patient data (age at death/ age at KD)	Sex	Cause of death	Gross pathology	Aneurysms	Organization/recanalization	Calcification
Murai 1989 (19)	1	23y/6y (susp.)	M	Ischemic heart failure	Multiple aneurysms; LCX occlusion; Scattered foci of myocardial fibrosis	+	+/- Fresh thrombus; Organized thrombus	+
Fineschi 1999 (20)	1	21y/3y	M	SCD	Small whitish patch on the anterior wall of the left ventricle; Calcified saccular aneurysms in the LAD and RCA; Significant stenosis distal to the aneurysm	+	+/- Organized thrombus	+
Orenstein 2012 (8)	1	22y/3y	F	MI	Significant luminal stenosis	–	-/-	–
Okura 2013 (21)	1	30y/4y	M	SCD	Calcified giant aneurysm; Segmental stenosis proximal and distal to the aneurysm; Bilateral pulmonary edema	+	+ (Nature unknown)	+
Shimizu 2015 (7)	2	22y/2y	M	SCD with chronic ischemic changes	Calcified aneurysm; Adjacent LAD of small caliber with partial occlusion	+	+/- Organized thrombus	+
		30y/6y(susp.)	M	MIS	LAD saccular aneurysm; RCA recanalized aneurysm; posterior LV wall infarct	+	+/+ Fresh thrombus; Organized thrombus	+
Gallego 2022 (17)	1	31y/2y	M	Cardiac arrest; hypoxic brain injury	Aneurysm with severe stenosis 5 cm scar on the posterolateral LV wall; Bilateral pulmonary edema and consolidation; Diffuse hypoxic-ischemic brain injury	+	+/- Organized thrombus	+

M, male; F, female; susp., suspected; MI, myocardial infarction; SCD, sudden cardiac death; y, year; LCX, left circumflex artery; LAD, left anterior descending artery; RCA, right coronary artery; LV, left ventricle; +, present; -, absent.

The pathological evolution of KD exhibits multidimensional characteristics in terms of both staging criteria and temporal boundaries. The “three-process” model suggests that these pathological processes may coexist and overlap within a single patient and are not strictly constrained by time; in contrast, classical studies typically stage the disease according to the number of days since onset (22). These differences in definitional emphasis are also related to the temporal distribution of autopsy sample acquisition across studies. Early KD research, predominantly based on Japanese cases, included a greater number of deaths during the acute phase, thereby providing detailed documentation of the transmural vascular destruction caused by NA. In contrast, more recent Western studies have focused on long-term cases occurring months to years after disease onset, revealing the luminal remodeling process driven by LMP (8, 22).

Notably, the vascular pathological evolution of KD represents a dynamic process driven by both individual factors and environmental triggers, exhibiting substantial phenotypic heterogeneity. Among these, therapeutic intervention plays a critical regulatory role in shaping disease progression. Evidence suggests that early administration of IVIG can effectively halt the progression of NA and preserve vascular structural integrity, thereby reducing the incidence of coronary artery abnormalities (8, 23). Furthermore, KD progression demonstrates marked age dependency. Infants younger than 6 months often present with atypical clinical features and have a significantly increased risk of developing coronary artery abnormalities (4). Genetic factors and ethnic disparities further contribute to this heterogeneity. The incidence of KD in Japan is approximately 10–20 times higher than that in Western countries, and the risk is significantly elevated among siblings and children of affected parents, often exhibiting familial clustering (24). Phenotypic heterogeneity in KD is also associated with the diversity of infectious triggers, including a wide range of viruses, bacteria, and environmental factors (25). However, multiple studies have indicated that infectious factors do not influence the progression of coronary artery lesions (26, 27). Fully accounting for phenotypic heterogeneity driven by host–environment interactions is essential for advancing precise diagnosis and personalized treatment strategies in KD.

### 2.2 Myocardial, pericardial, and valvular pathology

Beyond the coronary arteries, other cardiac structures are often involved in KD, including myocarditis, pericarditis, and valvulitis (**Supplementary Table 3**) (4, 14).

#### 2.2.1 Myocarditis

Myocarditis occurs more frequently in the acute phase of KD, and reports of myocardial biopsy performed early in the course of the disease indicate that the incidence of myocarditis is widespread (4). The main pathological features include myocardial interstitial edema and inflammatory cell infiltration predominantly composed of monocytes and neutrophils, whereas myocardial necrosis is relatively uncommon (28). Accordingly, KD-associated myocarditis is generally responsive to anti-inflammatory therapy, with rapid improvement, and cardiac dysfunction is typically transient (4, 29).

However, autopsy reports have documented cases of fulminant myocarditis in KD, leading to cardiogenic shock and sudden death. Studies have shown that its pathological features include diffuse myocardial necrosis and severe inflammatory cell infiltration (29). Fulminant myocarditis may contribute to the pathogenesis of KD shock syndrome (KDSS), which is characterized by refractory hypotension and hypoperfusion (4, 28). These findings highlight the need for heightened clinical vigilance when managing patients presenting with KDSS. Given the rapid progression of lesions and the delayed response to pharmacological therapy, early initiation of extracorporeal life support should be considered in clinical practice to avoid missing the opportunity for timely rescue due to sole reliance on drug therapy (29, 30).

#### 2.2.2 Pericarditis

Pericardial involvement is common in KD, although clinical manifestations are generally mild (4, 31). Approximately 3% of patients develop transient pericardial effusion during the disease course, which typically resolves within 5 weeks (28). Autopsy studies indicate that KD-associated pericarditis is characterized by edema of the epicardium (visceral pericardium) with prominent infiltration of lymphocytes and plasma cells (32). Inflammatory changes are often most pronounced in areas surrounding the affected coronary arteries (4, 32). In clinical practice, cardiac tamponade is rare but can be life-threatening when it occurs. It may present as part of a polyserositis syndrome or arise secondary to rupture of a coronary artery aneurysm (CAA) into the pericardial space. Tamponade associated with polyserositis typically occurs during the acute phase of KD, whereas that resulting from CAA rupture may occur at any stage of the disease, including years after the acute phase (28).

#### 2.2.3 Valvulitis

Valvular abnormalities are also observed in patients with KD and represent a manifestation of acute-phase pancarditis. The most common valvular involvement is mitral regurgitation (MR), which is primarily attributed to valvular edema and inflammatory cell infiltration. Echocardiographic studies have reported that approximately 27% of patients exhibit mild MR during the acute phase (28). This regurgitation generally resolves during follow-up; however, residual valvular dysfunction persists in a subset of patients, approximately 9%, and may occasionally progress to severe MR. In rare cases, rupture of the chordae tendineae can lead to rapid clinical deterioration and even death (28). Autopsy findings in KD patients with MR reveal nonspecific morphological changes of the cardiac valves, characterized by lymphocytic and plasma cell infiltration, marked fibrosis, and neovascularization, resulting in leaflet thickening and shortening of the chordae tendineae (32). In the case of persistent valvular abnormalities, the pathogenesis may be different, possibly due to valve dysfunction and papillary muscle dysfunction due to coronary ischemia (28).

### 2.3 Pathological changes in other systems

As a systemic vasculitis, KD is not confined to the heart but can involve multiple organ systems throughout the body (14). Although the severity and clinical manifestations are generally less pronounced than those of cardiovascular involvement, they may still result in clinically significant consequences and therefore warrant careful consideration.

#### 2.3.1 Respiratory system

Clinically, KD is often accompanied by prodromal respiratory symptoms, resulting in significant pathological changes in the respiratory system (33). It has been reported that 56% to 83% of patients with KD have a history of preceding respiratory disease (33). Autopsy studies have shown that the lungs of KD patients may exhibit interstitial pneumonia, alveolar macrophage infiltration, and pleural effusion (4, 34). Studies have shown that during the acute phase of KD, abundant IgA-producing plasma cells infiltrate the submucosal glands of the trachea and bronchi (35). Furthermore, unique intracytoplasmic inclusions (ICIs) have been observed in the bronchial ciliated epithelial cells of patients during the acute phase (9). Special staining confirmed the presence of RNA, and electron microscopy revealed virus-like particles (VLPs) within these inclusions (36, 37).

A recent study analyzed the plasmablast response in 12 children during the acute phase of KD and demonstrated that all subjects produced monoclonal antibodies capable of recognizing ICIs, predominantly of the IgA or IgG isotype. The ICI particles were primarily localized within medium-sized airways, such as the small bronchi and bronchioles, but were absent in the trachea, nasal mucosa, and peripheral bronchioles. This distribution may reflect the presence of site-specific pathogen receptors (38). The persistence of these inclusions, detectable months or even years after the acute phase, supports the hypothesis of a persistent infection within the respiratory tract (36). Based on the persistence of ICIs and their epidemiological features, a dual model of respiratory transmission has been proposed. The first involves community-level droplet or contact transmission driven by asymptomatic children, characterized by winter–spring seasonality. The second is a non-seasonal pathway involving persistent infection, with transmission to infants via household contacts such as parents or siblings (38). These findings are consistent with the hypothesis that the infectious agent of KD is a “novel” RNA virus and provide further support for the respiratory tract as a potential portal of entry.

#### 2.3.2 Digestive system

Common gastrointestinal manifestations of KD include hepatitis, diarrhea, vomiting, and gallbladder hydrops (4). Pathological studies have shown that gallbladder hydrops associated with KD corresponds to acute suppurative cholecystitis, with sterile bile cultures, suggesting that the inflammation is non-infectious in nature (39). Microscopic examination demonstrated severe neutrophilic infiltration involving the submucosa, muscularis, and serosal layers of the gallbladder wall (39). Liver involvement is also commonly observed in KD. Studies have reported the presence of IgA-producing plasma cells surrounding the intrahepatic bile ducts in approximately 60% of patients (35). In addition, autopsy findings indicate mild hepatocellular steatosis, slight hepatocyte swelling, and an increased number of Kupffer cells during the acute phase of KD (40). Pancreatic involvement has also been described in a subset of patients. Relevant studies have shown that IgA-producing plasma cells can be detected around the pancreatic ducts, occasionally accompanied by acinar atrophy and fibrosis (35). In light of evidence suggesting intestinal barrier dysfunction, some researchers have proposed the potential role of the “gut portal” as a triggering factor for inflammation (41).

#### 2.3.3 Urinary system

Urinary system involvement is relatively uncommon in KD, with clinical manifestations typically including sterile pyuria, hematuria, and proteinuria (34, 42). In KD patients with pyuria, urinary cells are predominantly mononuclear rather than neutrophilic. In addition, cytoplasmic inclusions have been identified within mononuclear cells in the urinary sediment (43, 44). The most common renal pathological finding in KD is tubulointerstitial nephritis (TIN), characterized by inflammatory cell infiltration of the renal interstitium (42). This has been supported by autopsy studies, including a case of atypical KD presenting with hematuria as the initial symptom, which demonstrated infiltration of lymphocytes, plasma cells, and eosinophils in the renal interstitium (34). Further studies have also confirmed the presence of IgA-producing plasma cells in the renal interstitium in nearly all KD autopsy cases (35). Moreover, as a systemic vasculitis, KD-associated inflammation may involve not only the coronary arteries but also the renal arteries, potentially leading to renal aneurysm formation or vascular stenosis (42).

#### 2.3.4 Nervous system

KD may also lead to neurological involvement. It may involve the meninges resulting in aseptic meningitis, which is a common neurological complication in the acute phase of KD (4). In patients undergoing lumbar puncture, approximately 30% show increased cell counts in the cerebrospinal fluid (CSF), predominantly monocytes, with generally normal glucose and protein levels (4, 45). In addition, a small proportion of patients may present with transient facial paralysis and sensorineural hearing impairment (4). Pathological investigations have further shown that, in fatal cases, approximately 50% of patients exhibit aseptic choroiditis or leptomeningitis. Histological examination demonstrates thickening of the leptomeninges due to fibroblastic proliferation, accompanied by diffuse infiltration of lymphocytes and monocytes (46). Within the brain parenchyma, marked perivascular and perineuronal edema is commonly observed, and some cases may exhibit edematous necrosis and neuronal degeneration (46). Although cerebral arteritis has also been reported, the severity of inflammation is generally much milder than that observed in the coronary arteries, possibly due to the relative paucity of perivascular connective tissue (46, 47). Furthermore, peripheral nerve involvement may be extensive, manifesting as generalized ganglionitis and neuritis affecting both sympathetic and parasympathetic ganglia. Histologically, infiltration of lymphocytes and plasma cells is observed, along with ganglion cell atrophy (46). These findings indicate that pathological alterations of the nervous system constitute an integral component of the systemic inflammatory process in KD.

#### 2.3.5 Lymphatic system

Cervical lymphadenopathy is one of the five principal diagnostic criteria for KD (4). Histologically, these lymph node lesions are typically nonspecific (4). However, clinically significant lymphadenopathy may mimic lymphoma, potentially leading to diagnostic delay (48). More detailed immunopathological studies have demonstrated that ICIs can be detected in macrophages within the peribronchial lymph nodes of KD patients. This finding is thought to trigger mucosal immune responses, resulting in the marked expansion of IgA-producing plasma cells and their subsequent widespread infiltration into non-lymphoid tissues, including the coronary arteries, pancreas, and kidneys (35, 36).

The non-cardiovascular pathological alterations in KD are of considerable significance. They not only reinforce the characterization of KD as a systemic vasculitis but also provide important insights into its potential association with infectious factors and possible portals of pathogen entry. **Table 2** summarizes the epidemiological features and major histopathological changes across different organ systems to facilitate a clearer overview. These findings suggest that certain commonly observed non-cardiovascular manifestations may serve as valuable adjunctive indicators in supporting the diagnosis of KD.

**Table 2:** Epidemiological characteristics and major pathological changes of other systemic involvement.

Involvement system	Clinical incidence	Clinical manifestations	Pathological changes at autopsy/biopsy
Respiratory System	56%~83% (33)	fever; cough; rhinorrhea; dyspnea (38, 47); pulmonary infiltrates (imaging) (49)	Trachea: periglandular IgA plasma cell aggregates (35); Bronchi: periglandular IgA plasma cell aggregates; ICIs in bronchial ciliated epithelia (9, 35–37); Lungs: interstitial/bronchopneumonia; occasional pulmonary arteritis (34, 49)
Digestive System	GI symptoms >61% (47); gallbladder fluid ~ 14% (39); liver dysfunction: 40~60% (4, 50); pancreatitis: rare (clinical) (4), 22%(pathological) (51)	diarrhea, vomiting, abdominal pain (GI symptoms); gallbladder hydrops; hepatitis; hepatosplenomegaly; pancreatitis	Gallbladder: acute suppurative cholecystitis (39); Liver: periportal IgA plasma cell infiltration (35), hepatocellular swelling (40); Pancreas: periductal IgA plasma cell infiltration; focal acinar atrophy and fibrosis (35)
Urinary System	sterile pyuria: 30%~75% (42); TIN: rare (clinical) (42), 25%~36% (pathological) (42)	sterile pyuria; hematuria; proteinuria; acute kidney injury	Urine sediment: mononuclear cells with inclusion bodies (43, 44); Renal interstitium: infiltration of lymphocytes, IgA plasma cells, and eosinophils (34, 35)
Nervous System	5.1% (52)	irritability; drowsiness (4, 51); facial nerve palsy; hearing loss (53)	Leptomeningeal inflammation (46); Facial nerve vasculitis (51, 53); Parenchymal edema (46); Extensive peripheral neuritis (46)
Lymphatic System	50%-75% (54)	Cervical lymphadenopathy (>1.5 cm) (4)	Peribronchial lymph nodes: ICIs within macrophages (37)

ICIs, intracytoplasmic inclusion bodies; TIN, tubulointerstitial nephritis; GI, gastrointestinal.

**Figure 1:**
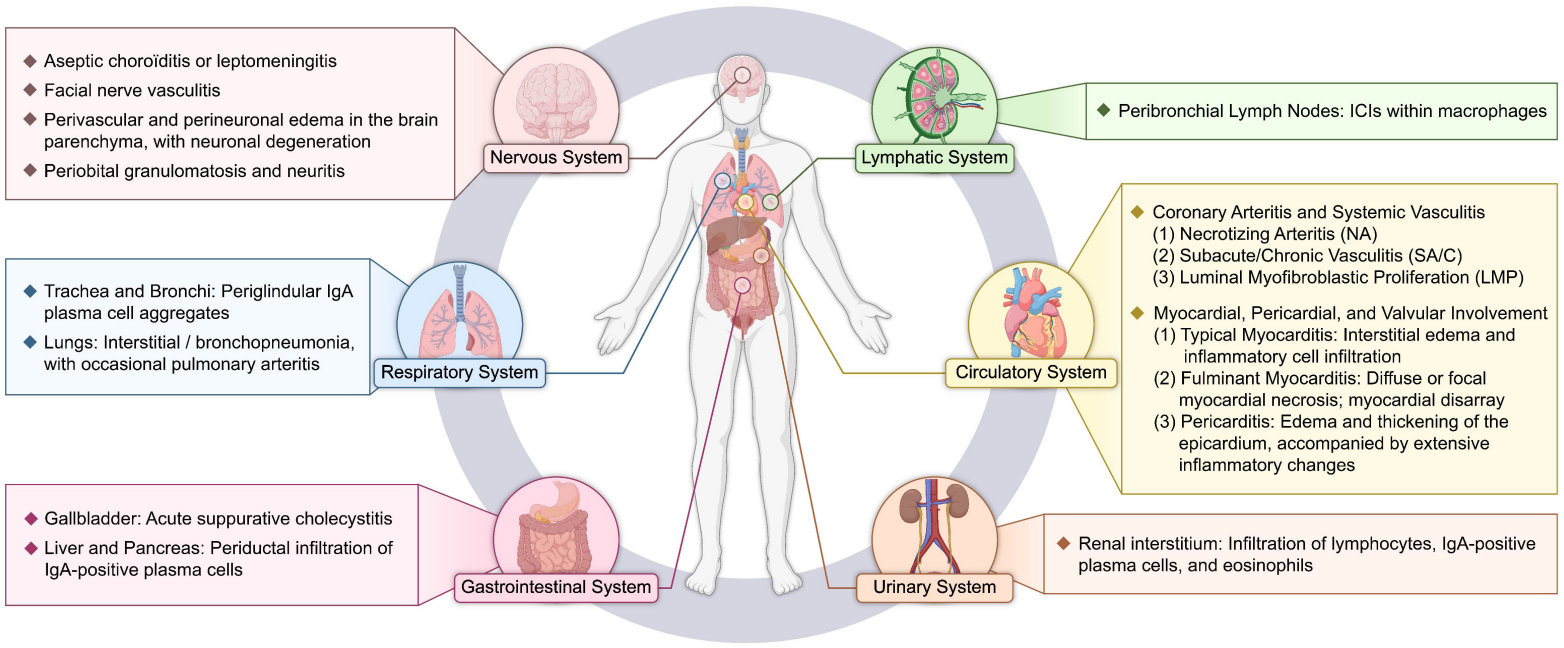
Schematic overview of the systemic pathological features of Kawasaki disease. ICI, intracytoplasmic inclusions.

The data presented in the table suggest that, although severe non-cardiovascular organ involvement is relatively uncommon in clinical practice, subclinical and occult inflammation is frequently observed. This observation highlights the pathological nature of KD as a systemic vasculitis that extensively involves small arteries and solid organs. Such widespread non-cardiovascular involvement provides important supportive evidence for the diagnosis of KD, particularly in the evaluation of incomplete KD. Current guidelines have identified sterile pyuria and elevated transaminase levels as key diagnostic indicators, prompting clinicians to recognize the potential risk of vasculitis at an early stage (4). **Figure 1** summarizes the pathological features of KD.

## 3 Molecular pathogenesis and clinical translation

### 3.1 Molecular mechanisms

#### 3.1.1 TLRs/NF-κB signaling pathway

**Figure 2:**
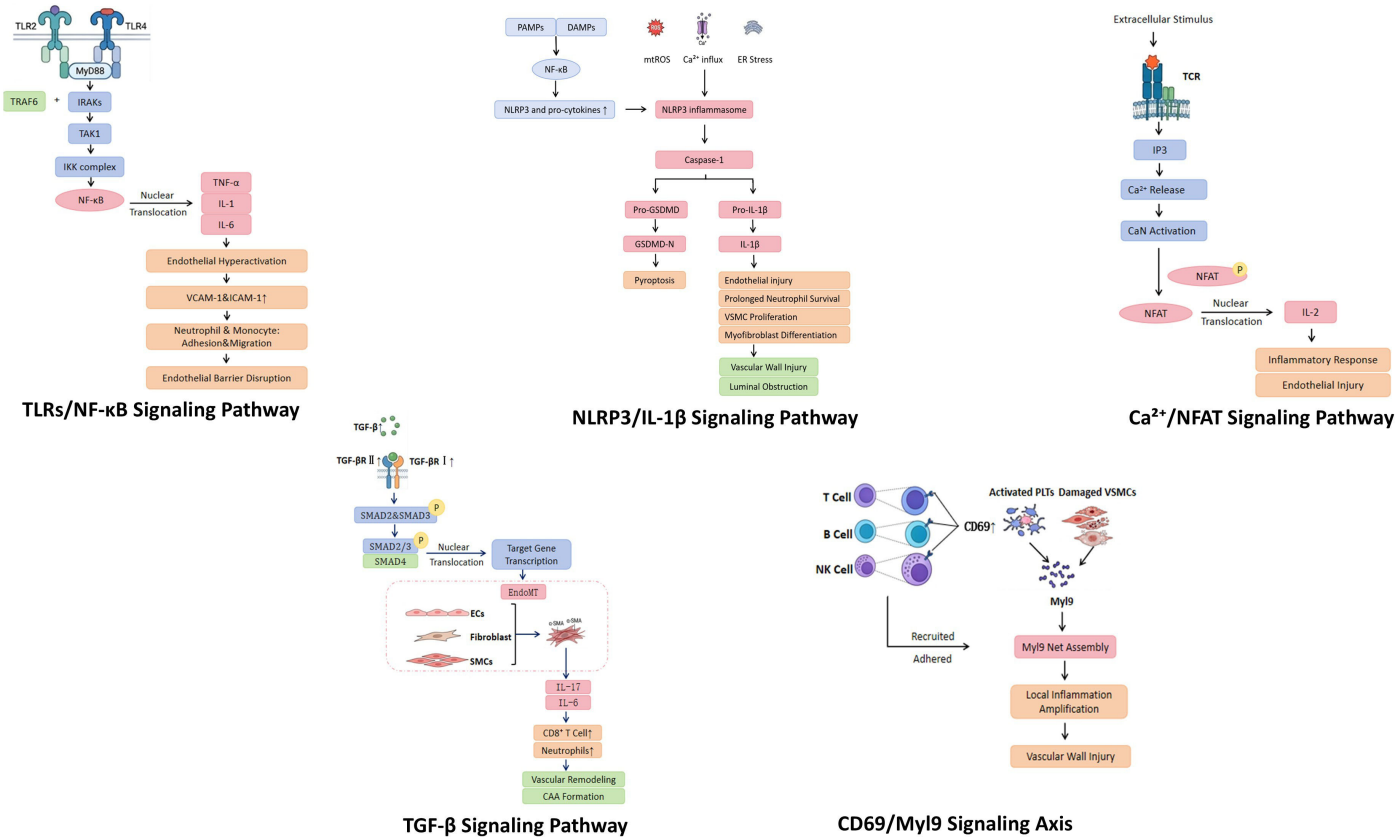
Key molecular signaling pathways involved in the pathogenesis of Kawasaki disease. TLRs, Toll-like receptors; NF-κB, nuclear factor kappa B; NLRP3, NOD-like receptor family pyrin domain-containing 3; IL-1β, interleukin-1 beta; Ca²^+^, calcium ion; NFAT, nuclear factor of activated T cells; TGF-β, transforming growth factor beta; Myl9, myosin light chain 9; CAA, coronary artery aneurysm; VSMC, vascular smooth muscle cell; PLT, platelet; EC, endothelial cell; EndoMT, endothelium-to-mesenchymal transition; VCAM-1, vascular cell adhesion molecule-1; ICAM-1, intercellular adhesion molecule-1; GSDMD, gasdermin D; MyD88, myeloid differentiation primary response 88; IRAKs, interleukin-1 receptor-associated kinases; TRAF6, TNF receptor-associated factor 6; TAK1, transforming growth factor beta-activated kinase 1; IKK, IκB kinase; TNF-α, tumor necrosis factor alpha; IL-6, interleukin-6; JNK, c-Jun N-terminal kinase; mtROS, mitochondrial reactive oxygen species; ER stress, endoplasmic reticulum stress; IP3, inositol 1,4,5-trisphosphate; CaN, calcineurin; TCR, T cell receptor; SMAD, mothers against decapentaplegic homolog; TGFBR, transforming growth factor beta receptor; α-SMA, alpha-smooth muscle actin; NK cell, natural killer cell.

**Table 3** and **Figure 2** summarize the cellular pathological changes and molecular mechanisms in KD. The pathogenesis of KD vasculitis is widely considered to be mediated by an abnormal host immune response triggered by as-yet-unidentified pathogens or environmental factors (55). Current evidence suggests that mucosal barriers and epithelial tissues, which are widely distributed throughout the body, serve as the first line of defense against external stimuli and act as the primary sites for sensing pathogenic signals and initiating innate immune responses. Upon recognition of danger-associated signals by Toll-like receptors (TLRs), particularly Toll-like receptor 2 (TLR2) and Toll-like receptor 4 (TLR4), expressed on mucosal epithelial cells and resident immune cells, intracellular signaling cascades are rapidly activated through adaptor proteins such as myeloid differentiation primary response 88 (MyD88). This process leads to the robust activation of the nuclear factor kappa B (NF-κB) signaling pathway (55, 56).

**Table 3:** Summary of cellular pathological evolution in KD.

Cell type	Tissue sampled/ Sampling time	Pathological changes	Biological marker	Clinical manifestations	References
Endothelial Cells	Coronary artery intima Acute phase: Approximately 7–9 days post-onset	Highly activated and edematous under the stimulation of cytokines (TNF-α, IL-1); regulation of adhesion molecules to mediate inflammatory cell migration.	TNF-α, IL-1, IL-6; TGF-β	Edema of the vessel wall; a critical initiating step for NA.	McCrindle 2017 (4) Orenstein 2012 (8) Qiu 2022 (57)
Smooth Muscle Cells	Coronary artery media From acute phase (12 days post-onset) to chronic phase (11 years post-onset)	Acute phase: Neutrophil-driven degeneration, necrosis, and lysis. Chronic phase: Phenotypic transformation driven by the TGF-β signaling pathway, converting into myofibroblasts.	NLRP3/IL-1β; TGF-β	Acute destruction leading to aneurysm formation; subsequent myofibroblast proliferation leading to luminal stenosis or occlusion.	Orenstein 2012 (8) Shimizu 2012 (16) Kuijpers 2003 (58)
Fibroblasts	Coronary artery adventitia Subacute/Chronic phase: Weeks to years’ post-onset	Activated into myofibroblasts; secretion of pro-inflammatory cytokines (e.g., IL-6, IL-7) and extracellular matrix components such as TN-C.	TGF-β	Induction of vessel wall fibrosis and remodeling.	Yokouchi 2018 (6) Orenstein 2012 (8) Shimizu 2012 (16)
Epithelial Cells	Bronchial, pancreatic, and renal ductal epithelium From acute phase (13 days post-onset) to chronic phase (7 years post-onset)	Formation of ICI (containing RNA and viral proteins); extensive infiltration of IgA plasma cells surrounding the cells.	ICI	Suggests the respiratory tract as a potential portal of entry; occurrence of extracardiac multi-organ involvement (e.g., interstitial nephritis, pancreatitis).	Rowley 2000 (35) Rowley 2008 (36) Watanabe 2013 (42)
Neutrophils	Coronary artery; myocardial interstitium Acute phase: Peak at 6–10 days post-onset	Early core infiltrating cells; release of proteases driving synchronized destruction of the vessel wall.	NLRP3/IL-1β	Driving NA; causing early diffuse myocardial injury.	Orenstein 2012 (8) Harada 2012 (59)
Macrophages	Coronary artery; myocardial interstitium Subacute phase: Prominent after 10 days, dominant after 20 days	Replacing neutrophil infiltration; involvement in granulomatous-like inflammation and subsequent tissue repair.	TLRs/NF-κB; NLRP3/IL-1β	Marking the transition from acute necrosis to reparative inflammation; involvement in long-term vascular remodeling.	Orenstein 2012 (8) Harada 2012 (59)
T Lymphocytes	Coronary artery and myocardial tissue Acute phase	CD8+ T cells are predominant; significant upregulation of CD69, binding with Myl9 released by activated platelets.	Ca2+/NFAT; CD69/Myl9	Exacerbation of vasculitis.	Orenstein 2012 (8) Brown 2001 (60) Kobayashi 2023 (61)
IgA Plasma Cells	Coronary artery wall; periepithelial ducts and glands Acute phase	Extensive infiltration of non-vascular epithelial ducts and surrounding glandular tissues.	/	Suggests potential respiratory entry of the pathogen; explains the etiology of non-cardiovascular systemic inflammation.	Rowley 2000 (35)

KD, Kawasaki disease; TNF-α, tumor necrosis factor alpha; IL, interleukin; NA, necrotizing arteritis; IVIG, intravenous immunoglobulin; TGF-β, transforming growth factor beta; TN-C, tenascin-C; ICI, intracytoplasmic inclusion bodies; Myl9, myosin light chain 9; NLRP3, NOD-like receptor family pyrin domain-containing 3; NF-κB, nuclear factor kappa B; TLRs, Toll-like receptors; Ca²^+^, calcium ion; NFAT, nuclear factor of activated T cells.

Activated NF-κB subsequently translocates to the nucleus, where it drives the transcription and release of numerous pro-inflammatory cytokines, including tumor necrosis factor alpha (TNF-α), interleukin-1 (IL-1), and interleukin-6 (IL-6), thereby contributing to an early cytokine surge (56, 62). These circulating inflammatory mediators primarily target coronary artery endothelial cells. Under sustained stimulation, endothelial cells become activated and upregulate the expression of endothelin, as well as adhesion molecules such as vascular cell adhesion molecule-1 (VCAM-1) and intercellular adhesion molecule-1 (ICAM-1) (62). VCAM-1 and ICAM-1 facilitate the adhesion and transmigration of inflammatory cells, including neutrophils and monocytes, thereby amplifying local inflammatory responses. This cascade ultimately disrupts endothelial barrier integrity and contributes to cardiovascular injury (62). Based on this molecular mechanism, the latest 2024 KD guidelines recommend infliximab, a TNF-α inhibitor targeting the NF-κB pathway, as an important therapeutic option, further supporting the translational relevance of this pathway in mitigating cardiovascular damage in KD (5).

#### 3.1.2 NLRP3/IL-1β signaling pathway

The activation of the NOD-like receptor family pyrin domain-containing 3 (NLRP3) inflammasome and the interleukin-1 beta (IL-1β) signaling axis plays a critical role in the initiation and progression of NA (63). The NLRP3 inflammasome is predominantly localized in innate immune cells, such as macrophages and neutrophils within the coronary artery wall. Its activation is driven by multiple cellular stress signals, including c-Jun N-terminal kinase (JNK)-mediated mitochondrial reactive oxygen species production, disruption of calcium homeostasis, and endoplasmic reticulum stress (63). Upon activation, the NLRP3 inflammasome promotes caspase-1 activation, which cleaves pro–IL-1β into its mature form, leading to the release of large amounts of IL-1β into the extracellular space (64). Concurrently, caspase-1 also cleaves gasdermin D, triggering pyroptotic cell death (64).

Elevated levels of IL-1β act as a key mediator of vascular wall injury. This cytokine not only enhances endothelial damage and increases vascular permeability but also prolongs the survival of locally accumulated neutrophils, thereby exacerbating vascular injury. In parallel, IL-1β promotes abnormal proliferation of vascular smooth muscle cells and their differentiation into myofibroblasts, accompanied by increased production of matrix metalloproteinases (MMPs), ultimately contributing to luminal narrowing (63). Given the marked activation of the IL-1 pathway during the acute phase of KD and its role in driving cardiovascular injury, the recombinant IL-1 receptor antagonist anakinra has demonstrated promising clinical efficacy, particularly in alleviating fever and inflammatory manifestations in patients with IVIG-resistant KD (5).

#### 3.1.3 Ca²^+^/NFAT signaling pathway

With the shift of the immunopathological mechanisms of KD from innate to adaptive immunity, sustained activation of the Ca²^+^/nuclear factor of activated T cells (NFAT) signaling pathway is considered a key driver of abnormal T lymphocyte responses (24, 63). Upon stimulation, the T cell receptor (TCR) promotes the production of inositol 1,4,5-trisphosphate (IP3), leading to a rapid increase in intracellular calcium ion (Ca²^+^) concentration and activation of calcineurin (CaN) (24, 65). CaN subsequently facilitates the dephosphorylation and nuclear translocation of NFAT, thereby inducing the transcription of pro-inflammatory cytokines such as interleukin-2 (IL-2) (24, 65).

Current evidence suggests that this intracellular signaling pathway is markedly dysregulated and pathologically overactivated in KD, potentially associated with genetic defects. As a key negative regulator of the Ca²^+^/NFAT signaling pathway, inositol 1,4,5-trisphosphate 3-kinase C (ITPKC) normally suppresses excessive activation of T and B cells. However, C-allele variants may impair this inhibitory function, leading to aberrant amplification of intracellular signaling and ultimately driving the characteristic immune hyperreactivity of KD (65). Overactivated T lymphocytes produce multiple cytokines that contribute to inflammatory responses and vascular endothelial injury, potentially resulting in aneurysm formation (66). Huang et al. further reported that the forkhead box O4 (FOXO4)/NFAT2 axis is associated with endothelial homeostasis and the development of cardiovascular inflammation (67).

Targeting this pathological process, cyclosporine A, a specific T-cell inhibitor that effectively blocks the calcium-driven calcineurin-NFAT pathway, has currently also been explored as a clinical therapeutic option for KD.

#### 3.1.4 TGF-β signaling pathway

The transforming growth factor beta (TGF-β) signaling pathway exerts bidirectional regulatory effects in vasculitis among KD patients (16). During disease progression, its aberrant activation is driven by multiple synergistic mechanisms. Studies indicate that host-related factors, including genetic susceptibility and markedly increased expression of TGF-β ligands and its receptor transforming growth factor beta receptor 2 (TGFBR2) in KD-damaged vessels, are key contributors to pathway overactivation (16). This aberrantly activated TGF-β signaling pathway promotes the endothelium-to-mesenchymal transition (EndoMT) process (16). Through this process, endothelial cells, fibroblasts, and smooth muscle cells can transdifferentiate into myofibroblasts, accompanied by loss of lineage-specific markers and acquisition of a phenotype characterized by high expression of α-smooth muscle actin (α-SMA) (16). Studies have demonstrated that, in human KD pathological specimens, abundant myofibroblasts with high α-SMA expression but lacking smoothelin are present within the thickened arterial intima (16). Furthermore, evidence from human autopsy specimens indicates that the aberrant migration of these myofibroblasts not only disrupts endothelial barrier integrity but also promotes the secretion of pro-inflammatory cytokines, including IL-17 and IL-6 (16). This pro-inflammatory microenvironment further recruits large numbers of human leukocyte antigen-DR (HLA-DR)-expressing activated CD8^+^ T cells and neutrophils, facilitating deep infiltration into the arterial wall. This cascade ultimately results in irreversible structural remodeling, vessel wall thickening, and the development of severe lesions such as CAA (16).

#### 3.1.5 CD69/Myl9 signaling axis

During the acute phase of KD vasculitis, the immune system is highly activated. Analyses of peripheral blood from KD patients have demonstrated that the transmembrane protein CD69 is markedly upregulated on various immune cells, including T lymphocytes, B lymphocytes, and natural killer (NK) cells (61). Similarly, in mice injected with Lactobacillus cell wall extract (LCWE), inflammatory cells with high CD69 expression were observed within the local microenvironment of diseased coronary arteries (61).

Concomitant with immune cell activation, plasma from acute-phase KD patients exhibits a pronounced increase in myosin light chain 9 (Myl9) (61). Notably, postmortem histopathological examination of KD patient coronary arteries shows abnormally high Myl9 expression within thrombi formed during the acute phase, as well as in thickened intimal and adventitial layers (61). Integrating findings from *in vitro* human experiments and animal model studies, this aberrant Myl9 release may result from excessive platelet activation or injury to vascular smooth muscle cells (61). Extracellular Myl9 aggregates into a distinct reticular structure, which recruits and adheres CD69-expressing inflammatory cells to the inflamed vascular wall. This mechanism amplifies local inflammation and accelerates vascular wall damage, contributing to the pathogenesis of KD vasculitis (61).

### 3.2 Genetic susceptibility

Genetic factors influence both disease susceptibility and severity by modulating immune responses. To date, genome-wide association studies (GWAS) have identified multiple single nucleotide polymorphisms (SNPs) in immune-related genes, including ITPKC, caspase 3 (CASP3), B lymphoid tyrosine kinase (BLK), CD40, and ORAI calcium release-activated calcium modulator 1 (ORAI1). These variants not only significantly increase disease risk but are also closely associated with the development of CAA and resistance to IVIG therapy (68).

For example, within the calcium signaling pathway, genetic variants in ITPKC, ORAI1, and solute carrier family 8 member A1 (SLC8A1) promote activation of NLRP3 inflammasome and production of IL-1β, thereby exacerbating vascular inflammation (55, 68). In terms of immune receptors and related pathways, variants in Fc gamma receptor IIa (FCGR2A) alter its binding affinity for IgG, affecting immune complex phagocytosis and leading to abnormal amplification of pro-inflammatory signaling pathways (68). Overactivation of immune cells within the CD40/CD40 ligand (CD40L) pathway further contributes to inflammation and vascular injury (68). In addition, CASP3 variants increase disease susceptibility by disrupting the regulation of T cell activation (68).

### 3.3 Clinical translation

The current standard treatment for KD consists of high-dose IVIG in combination with aspirin. Evidence indicates that administering IVIG within the first 10 days of disease onset reduces the risk of coronary arteritis and aneurysm formation from approximately 30% to 5–7% (55). While most patients respond favorably to IVIG, up to 20% remain resistant to initial therapy and are consequently at elevated risk for coronary artery complications (55).

For these patients, targeted interventions informed by specific signaling pathways and genetic factors provide critical guidance for precision treatment and clinical translation. For example, tumor necrosis factor-alpha (TNF-α) inhibitors such as infliximab can mitigate vascular damage by blocking endothelial activation and immune cell adhesion and migration, with studies demonstrating reduced fever duration and alleviation of anemia in IVIG-resistant patients (5). Targeting the Ca²^+^/NFAT signaling pathway, cyclosporine A suppresses IL-2 production and T-cell activation, thereby decreasing coronary artery lesions (5, 55). In addition, overactivation of the NLRP3/IL-1βpathway contributes to acute-phase NA, and multiple case reports indicate that the IL-1 receptor antagonist anakinra can effectively treat IVIG-resistant KD patients (5, 55). Concurrently, therapies such as tocilizumab, which targets the IL-6 pathway, and cyclophosphamide, which inhibits aberrant immune cell proliferation by blocking DNA replication, are increasingly used in patients unresponsive to multiple treatments (5). Importantly, candidate mechanisms such as TGF-β–related remodeling pathways and the CD69–Myl9 axis, as well as Tenascin-C (TN-C) as a potential biomarker, still require further validation. Before being applied to risk stratification or therapeutic decision-making, prospective and comparative studies in independent cohorts are needed.

For high-risk patients with established large coronary aneurysms, anticoagulation management is critical alongside active anti-inflammatory therapy. Currently, in addition to traditional warfarin or low molecular weight heparin, direct oral anticoagulants (DOACs) have also emerged as a novel anticoagulation option for this patient population (5). Looking ahead, targeted drug delivery technologies hold considerable promise for clinical translation, but their application in Kawasaki disease still requires further investigation and validation.

## 4 Discussion

Current evidence indicates that although KD is clinically defined as a systemic vasculitis affecting small- to medium-sized arteries, its primary and most significant lesions are concentrated in the coronary arteries. Previous studies have shown that although the inflammatory progression in large arteries, such as the aorta and coronary arteries, follows a broadly similar course, the severity of inflammation differs markedly between these vessels (69). Severe inflammation in the coronary arteries leads to destruction of the vessel wall and subsequent formation of CAA, whereas inflammation in the aorta is generally milder, and structural damage to the aortic wall is relatively uncommon (69). This discrepancy may be related to the anatomical characteristics of the coronary arteries. It has been proposed that vessels predisposed to aneurysm formation are typically muscular arteries characterized by the presence and distribution of vasa vasorum (70). Accordingly, coronary arteries, which have a relatively abundant vasa vasorum network, may exhibit unique susceptibility to injury. Experimental studies in mouse models have demonstrated that coronary arteries are highly dependent on the vasa vasorum for oxygen supply to the outer and medial layers of the vessel wall (70). During KD, the vasa vasorum undergo pronounced inflammation and edema, leading to ischemia and hypoxia of the vascular media. This process compromises the structural integrity of the coronary arterial wall, rendering it more fragile and ultimately predisposing to aneurysm formation (70). In addition, the “endothelial heterogeneity” hypothesis has been proposed, suggesting that although endothelial cells line the entire vascular system, they exhibit site-specific differences in the expression of chemokines, adhesion molecules, and other surface markers (8). Accordingly, it has been speculated that pathogenic factors associated with KD may be more highly expressed or preferentially interact with endothelial cells in the coronary arteries, whereas their expression or interaction in other vascular beds is relatively limited. This differential distribution may further contribute to the heightened susceptibility of coronary arteries to injury (8).

It is noteworthy that the pathological hallmarks of KD are not restricted to the coronary arteries but rather manifest as ‘pancarditis’ and systemic multisystem involvement. Within the heart, the core lesions of the acute phase are characterized by myocardial interstitial edema, inflammatory cell infiltration, inflammation of the endocardium and valves (particularly the mitral valve), and pericardial effusion. Beyond the cardiovascular system, pathological evidence indicates varying degrees of alterations across the respiratory, digestive, urinary, and nervous systems. The observed pathological heterogeneity across these organs not only highlights the systemic vasculitic nature of KD but also suggests a potential link to its underlying infectious triggers and site-specific immune response patterns.

This distinctive pathological basis partly explains the preferential involvement of the coronary arteries in KD and suggests a potential increase in long-term cardiovascular risk among affected patients, posing important challenges for long-term management. Evidence from long-term autopsy studies indicates that KD may represent a lifelong condition, with sequelae that can remain clinically silent for decades (7, 17, 71, 72). Autopsy findings further reveal that in a substantial proportion of adult sudden deaths attributed to myocardial infarction or other cardiac events, the underlying pathology can be traced back to childhood KD. These cases are characterized by giant CAAs, severe luminal stenosis, organized thrombi, and extensive calcification (7, 8, 17, 71, 72).

These findings underscore the importance of lifelong cardiovascular follow-up in patients with KD. Although large-scale longitudinal cohort studies directly quantifying the long-term risk of adult cardiovascular events following childhood KD remain limited, accumulating subclinical evidence suggests that KD may induce systemic and persistent vascular dysfunction after disease onset (73, 74). Studies have demonstrated that patients with a history of KD exhibit decreased flow-mediated dilatation (FMD), alongside elevated pulse wave velocity (PWV) and arterial stiffness index (73). Furthermore, at the structural level, related research indicates that the carotid intima-media thickness (CIMT) in these patients is increased compared to that in healthy populations (74).

In addition, the early vascular damage caused by KD is highly likely to act synergistically with later risk factors, such as dyslipidemia and hypertension, thereby increasing the risk of sudden death in adult KD patients. Therefore, long-term monitoring of vascular health and lipid profiles is crucial. Particularly for high-risk populations with previous coronary artery lesions, timely risk assessment and intervention through regular ultrasound follow-ups are of great clinical significance (73, 74).

The necessity of long-term follow-up in KD largely arises from the fact that its vascular pathological progression does not follow a single, fixed pattern but instead exhibits marked clinical and phenotypic heterogeneity. Variations in factors such as age at onset, ethnicity, and infectious triggers reflect, at a macro level, the interaction between environmental exposures and host genetic susceptibility. Advances in molecular pathology and genetics have further demonstrated that the pathogenesis of KD is not driven by a single factor, but rather by a complex network orchestrated by host genetic polymorphisms and core molecular pathways. On the one hand, polymorphisms in specific genes underpin individual susceptibility; on the other hand, multiple classical signaling pathways—including TLRs/NF-κB, NLRP3/IL-1β, Ca²^+^/NFAT, TGF-β, and CD69/Myl9—directly mediate the initiation and progression of vasculitis.

At present, the widespread use of IVIG therapy has significantly reduced the incidence of coronary artery complications in KD. However, further investigation into the etiology of the disease remains crucial. At the same time, the majority of KD patients without CAA have been confirmed to still have coronary artery vasculitis (18). Therefore, prospective studies are needed to determine whether there are hidden cardiovascular risks in the long term and to improve the comprehensive cognition and long-term management of KD.

By integrating classic autopsy studies with contemporary molecular pathological findings, this review summarizes the pathological basis of coronary artery involvement and discusses the pathological heterogeneity and clinical significance of cardiac and extracardiac organ involvement in KD. Future prospective studies should place greater emphasis on identifying occult risks in patients without CAA. Notably, with the rapid advancement of high-throughput technologies such as single-cell sequencing and spatial transcriptomics, an increasing number of research groups are shifting toward the direct analysis of human pathological specimens, moving beyond exclusive reliance on animal models to further elucidate the etiology and pathogenesis of KD. Although such in-depth, human tissue–based translational research requires time to accumulate robust datasets, investigations into the key molecular pathways underlying chronic vascular inflammation and structural remodeling remain of critical clinical importance. Mechanistic breakthroughs in this area may facilitate the identification of KD-specific molecular targets and provide new insights into precision prevention and long-term management of the disease.

## 5 Limitations

This review has several limitations. First, the pathological evidence base is constrained by the limited availability of contemporary human specimens. KD exhibits marked geographic and ethnic disparities in incidence, with East Asian countries representing high-prevalence regions; for example, the annual incidence among children under 5 years of age in Japan approaches 359 per 100,000, whereas in North America it is approximately 25 per 100,000 (4, 75). Despite these differences, the marked decline in acute-phase mortality, particularly in medically advanced regions where case fatality rates are typically below 0.1% (4), has substantially reduced opportunities for obtaining autopsy material. In addition, limited cultural acceptance of autopsy in many regions further restricts access to high-quality pathological specimens. As a result, much of the current understanding of KD pathology continues to rely on retrospective autopsy studies. This dependence may limit the generalizability of findings to the contemporary era, particularly in the context of widespread IVIG use, which may have altered patterns of pathological progression. Therefore, further validation using biopsy-derived specimens and emerging technologies is warranted.

## 6 Conclusion

KD is a systemic vasculitis with coronary arteries as the principal pathological target. Human pathology supports a dynamic progression from acute inflammatory injury to chronic vascular remodeling, which underlies aneurysm formation, stenosis, thrombosis, and late ischemic events.

Cardiac and extra-cardiac pathological findings further highlight KD as a multisystem disease. These findings not only aid in the early identification of KD particularly in atypical cases, but also play a crucial role in improving patients’ long-term systemic prognosis. Meanwhile, the increasingly elucidated core signaling pathways and genetic susceptibility loci are emerging as crucial breakthroughs for future precision targeted therapies in Kawasaki disease.

Future work should focus on prospective pathology-linked studies in the IVIG era, long-term vascular remodeling in patients with and without CAA, molecular dissection of stromal-immune interactions, and integrative multi-omics research to further elucidate the mechanisms of KD and support precision risk assessment and targeted therapy.
