# Supplementary material for: Human pathological findings in Kawasaki disease: a narrative review of autopsy and biopsy evidence
Source: Front Immunol. 2026 May 7;17:1798378. doi: 10.3389/fimmu.2026.1798378 (PMC13189798; doi:10.3389/fimmu.2026.1798378)
Supplement: Supplementary file 1 [file DataSheet1.docx]

**Supplementary Table 1. Summary of Human Pathological Studies on KD from Early to Contemporary Research.**

| Reference  (Author, Year) | SampleSize  (N) | Patient Characteristics | Disease Stage | Key Findings |
| --- | --- | --- | --- | --- |
| **Classic autopsy study** | | | | |
| Fujiwara & Hamashima  (1978) (1) | 20 | Age: 3 mo–5 yr  Sex: 14M / 6F | NI | Cardiac pathological staging: established the classic stage I–IV staging system; explicitly identified that the vasculitis originates from microvascular involvement |
| Amano (1980) (2) | 37 | Age: 3 mo–6 yr  Sex: 29M / 8F | 10 d–yr | Systemic pathological features: detailed analysis of various organs established the essence of KD as systemic vasculitis |
| Takahashi (2012)(3) | 380 | Pediatric vasculitis:380  (KD: n=198)  Age: <15 yr | NI | Epidemiological pathology: KD has replaced PN as the primary vasculitis in children |
| **Study on Histopathological Features and Mechanisms** | | | | |
| Orenstein (2012)(4) | 41 | Autopsy (n=32)  Cardiac transplantation (n=8)  CAA resection (n=1)  Age: 2 mo–22 yr  Male predominance | 10 d–19 yr | Defined the "Three Linked Vasculopathic Processes":  necrotizing arteritis,  subacute/chronic vasculitis,  luminal myofibroblastic proliferation |
| Harada  (2012)(5) | 29 | Age: 2 mo–9 yr  Sex: 19M / 10F | 6d–39d | Myocarditis characteristics: universal histological myocarditis in fatal acute-phase cases, characterized by inflammatory cell infiltration and interstitial edema |
| Sato  （2021）(6) | 37 | Acute phase (n=19)  Late phase (n=18)  Including 27 with CAA | NI | Aortitis present in 90% of cases |
| **Pathological Studies on Pathogenesis and Portal of Entry** | | | | |
| Rowley  （2000）(7) | 28 | KD Group (n=18)  (Age: 3 mo–10 yr;  Sex: 10M/8F)  Control Group (n=10) | 5d–28d | Demonstrated IgA plasma cell infiltration in the respiratory tract, pancreas, kidney, and coronary arteries, supporting the hypothesis of a respiratory portal of entry and systemic dissemination |
| Rowley  (2008) (8) | 34 | KD Group (n=7)  (Age:5mo–24yr; Sex:3M/3F/1ND)  Control Group (n=27) | 2 mo–21 yr | Evidence of persistent infection: identified RNA-containing ICI in ciliated bronchial epithelium in 86% of late cases (vs. 26% in controls), suggesting persistent viral infection |
| **Pathological Studies on Fatal Cardiovascular Events and Severe Complications** | | | | |
| Visi  （2023）(9) | 117 | Sex: 86M/20F/11NA  Age: 69.2% < 20 yr  (Systematic Review) | NI | AMI (41.03%) and CAA rupture (11.97%) identified as the leading causes of death in KD |
| Shimizu（2015）(10) | 10 | New Case 1: 22M  New Case 2: 30M Retrospective Cases: 17–30 yr | 15–26 yr | Affected coronary arteries are characterized by thrombus organization and recanalization, calcification, and severe intimal thickening caused by LMP. This constitutes the anatomical basis for silent ischemia and sudden death in adulthood |

*Abbreviations:* M, male; F, female; NI, no information; ND, no data; d, day; mo, month; yr, year; KD, Kawasaki disease; PN, polyarteritis nodosa; CAA, coronary artery aneurysm, ICI, intracytoplasmic inclusions; AMI, acute myocardial infarction; LMP, luminal myofibroblastic proliferation.

**Supplementary Table 2. Summary of Major Pathological Changes in KD Vasculitis.**

| **Pathological Stage** | **Timing & Nature** | **Infiltrating Cells** | **Pathological Features** | **Clinical Outcomes** |
| --- | --- | --- | --- | --- |
| **NA** | Within 2 weeks of fever onset;  Synchronous, self-limiting | Neutrophils | Synchronous neutrophilic infiltration; sequential destruction from the lumen to the adventitia. | Saccular aneurysms (rupture, acute thrombosis) |
| **SA/C** | Starting >2 weeks after fever onset; persists for months to years;  Asynchronous, progressive | Lymphocytes,  plasma cells, eosinophils,  (few macrophages) | Asynchronous infiltration starting from adventitia or perivascular tissue | Fusiform/saccular aneurysms;  progressive luminal stenosis |
| **LMP** | Starting >2 weeks after fever onset; persists for months to years  Asynchronous, progressive | Myofibroblasts | Progressive intimal thickening and luminal stenosis/occlusion | Thrombus organization (recanalization);  extensive calcification;  fibrotic intimal thickening |

*Abbreviations:* NA, necrotizing arteritis; SA/C, subacute/chronic vasculitis; LMP, luminal myofibroblastic proliferation.

**Supplementary Table 3. Summary of Cardiac Complications in KD.**

| Type of Complication | Pathological Changes | Clinical Manifestations | Causes of Death |
| --- | --- | --- | --- |
| Typical myocarditis | Myocardial interstitial edema; inflammatory cell infiltration dominated by mononuclear cells and neutrophils. | Common in the early stage of KD; tachycardia, gallop rhythm, and increased precordial impulse. | Severe arrhythmia, congestive heart failure, etc. |
| Fulminant myocarditis | Diffuse or focal myocardial necrosis; disorganized myocardial fibers; severe infiltration of neutrophils, mononuclear cells, and macrophages. | Refractory hypotension and hypoperfusion. | Cardiogenic shock, sudden death. |
| Pericarditis | Epicardial edema and thickening, accompanied by massive infiltration of lymphocytes and plasma cells. | Transient pericardial effusion; mostly mild and asymptomatic. | Acute cardiac tamponade. |
| Valvulitis | Valvular edema and inflammatory cell infiltration. | MR; 9% of patients experience persistent residual valvular dysfunction. | Chordae tendineae rupture leading to acute severe MR, resulting in rapid clinical deterioration and death. |

*Abbreviations:* KD, Kawasaki disease; MR, mitral regurgitation.

**References**

1. Fujiwara H, Hamashima Y. Pathology of the heart in Kawasaki disease. Pediatrics. (1978) 61:100-7.

2. Amano S, Hazama F, Kubagawa H, Tasaka K, Haebara H, Hamashima Y. General pathology of Kawasaki disease. On the morphological alterations corresponding to the clinical manifestations. Acta Pathol Jpn. (1980) 30:681-94.

3. Takahashi K, Oharaseki T, Yokouchi Y, Yamada H, Shibuya K, Naoe S. A half-century of autopsy results-incidence of pediatric vasculitis syndromes, especially Kawasaki disease. Circ J. (2012) 76:964-70. doi:10.1253/circj.cj-11-0928

4. Orenstein JM, Shulman ST, Fox LM, Baker SC, Takahashi M, Bhatti TR, et al. Three linked vasculopathic processes characterize Kawasaki disease: a light and transmission electron microscopic study. PLoS One. (2012) 7:e38998. doi:10.1371/journal.pone.0038998

5. Harada M, Yokouchi Y, Oharaseki T, Matsui K, Tobayama H, Tanaka N, et al. Histopathological characteristics of myocarditis in acute-phase Kawasaki disease. Histopathology. (2012) 61:1156-67. doi:10.1111/j.1365-2559.2012.04332.x

6. Sato W, Yokouchi Y, Oharaseki T, Asakawa N, Takahashi K. The pathology of Kawasaki disease aortitis: a study of 37 cases. Cardiovasc Pathol. (2021) 51:107303. doi:10.1016/j.carpath.2020.107303

7. Rowley AH, Shulman ST, Mask CA, Finn LS, Terai M, Baker SC, et al. IgA plasma cell infiltration of proximal respiratory tract, pancreas, kidney, and coronary artery in acute Kawasaki disease. J Infect Dis. (2000) 182:1183-91. doi:10.1086/315832

8. Rowley AH, Baker SC, Shulman ST, Garcia FL, Fox LM, Kos IM, et al. RNA-containing cytoplasmic inclusion bodies in ciliated bronchial epithelium months to years after acute Kawasaki disease. PLoS One. (2008) 3:e1582. doi:10.1371/journal.pone.0001582

9. Visi G, Spina F, Del Duca F, Manetti AC, Maiese A, La Russa R, et al. Autoptic findings in cases of sudden death due to Kawasaki disease.Diagnostics. (2023) 13:1831. doi:10.3390/diagnostics13111831

10. Shimizu C, Sood A, Lau HD, Oharaseki T, Takahashi K, Krous HF, et al. Cardiovascular pathology in 2 young adults with sudden, unexpected death due to coronary aneurysms from Kawasaki disease in childhood. Cardiovasc Pathol. (2015) 24:310-6. doi:10.1016/j.carpath.2015.02.006
